# Spinoplastic Surgery: A Review of Techniques, Indications, and Optimal Patient Selection

**DOI:** 10.3390/brainsci15070705

**Published:** 2025-06-30

**Authors:** Daniel Vernik, Camryn Payne, Krishna Sinha, Casey Martinez, Walter Nicholas Jungbauer, Jonathan L. Jeger, Michael Bohl, Alexander E. Ropper, Sebastian Winocour, Edward Reece

**Affiliations:** 1 Mayo Clinic Alix School of Medicine, Arizona Campus, Phoenix, AZ 85054, USA; vernik.daniel@mayo.edu (D.V.);; 2 Division of Plastic and Reconstructive Surgery, Mayo Clinic, Phoenix, AZ 85054, USA; 3 Carolina Neurosurgery & Spine Associates, Charlotte, NC 28204, USA; 4 Department of Neurosurgery, Baylor College of Medicine, Houston, TX 77030, USA; 5 Department of Plastic Surgery, Baylor College of Medicine, Houston, TX 77030, USA

**Keywords:** spinoplastics, complex spinal reconstruction, pseudoarthrosis, vascularized bone graft, spinal surgery

## Abstract

**Background/Objectives:** Spinoplastic surgery is an emerging multidisciplinary field developed to address and reduce the complication of pseudoarthrosis following complex spinal reconstructions. While the number of spinal fusion procedures continues to rise every year, fusion failure rates remain as high as 40%. Although pseudoarthrosis may not always manifest clinically, it remains a leading cause of persistent pain and need for subsequent revision surgeries. The multidisciplinary collaboration between spine and plastic surgeons in spinoplastic surgery has therefore emerged as a proactive strategy aimed at preventing complications, particularly in patients identified as high-risk for pseudoarthrosis. As the patient population expands and spinoplastic surgery continues to evolve, refining patient selection criteria becomes essential for achieving optimal surgical outcomes. This review aims to provide a comprehensive overview of recent advancements in spinoplastic surgery, highlighting current indications, surgical techniques, recent case reports, and strategies for identifying suitable candidates. **Methods:** We performed a narrative review of English language literature through April 2025. Spinoplastic case reports and case series published within the last 20 years were included in the review. **Results:** Indications for use of a spinoplastic approach clustered into prior fusion failure, extensive oncologic resection, severe spinal deformity, procedures requiring extensive spinal involvement, and/or patients at risk for impaired bone healing. Succesful radiographic union and improvement of symptoms were widely reported across all 9 case reports/series. **Conclusions:** Although evidence is presently limited, spinoplastic surgery appears to achieve high bone graft fusion rates with acceptable morbidity and functional improvement in a carefully selected group of high-risk spinal reconstruction patients. Still, larger prospective studies are warranted to refine patient selection and validate functional benefit.

## 1. Introduction

Spinoplastic surgery is an emerging multidisciplinary specialty that combines the unique skillsets of neurosurgery, orthopedic, and plastic and reconstructive surgeons to rise to the challenge of enhancing reconstructive outcomes in complex spinal surgery patients [1].

Spinoplastic surgery offers a unique solution for these patients by leveraging vascularized bone grafts (VBGs) to supplement traditional spinal fusion techniques [1]. VBGs are pedicled musculo-osseus grafts perfused by Sharpey’s fibers and periosteal feeding vessels, bringing reliable blood flow to the spinal defect in order to promote wound healing, antimicrobial defense, and structural integrity [2,3]. VBGs offer a superior alternative to other options on the spinal reconstructive ladder, such as free flaps, which are associated with heightened donor site morbidity as well as increased operative times compared to VBGs due to the need for microscope use [2,4,5,6]. There are six primary types of VBGs for spinal recostruction, each pedicled on neughboring vasculature: the iliac crest, rib, scapula, clavicle, spinous process, and occiput [1,2]. Ultimately, spinoplastic surgery can be defined as a multidisciplinary approach towards complex spinal surgery that utilizes VBGs for augmentation of outcomes.

Spinal arthrodesis is a surgical procedure indicated for a variety of conditions such as degenerative disc disease, spondyloses, oncologic resections, and trauma, and involves fusion of the vertebrae to stabilize the spine [7]. However, these procedures can have significant complications with pseudoarthrosis (failed bony fusion) rates reported as high as 40% and reoperation rates up to 19% within four years [7,8,9,10]. This is a devastating complication due to failure of the intended bony fusion that can lead to chronic axial and radicular pain, neurologic symptoms, and need for reoperation [7,11,12,13,14,15]. Due to its ability to promote superior wound healing and structural integrity to spinal defects, a spinoplastic surgical approach offers a valuable alternative surgical option for patients who are at heightened risk of pseudoarthrosis. However, as the patient population grows and the field continues to advance, patient selection criteria must be refined to optimize outcomes [16,17]. Currently, the indicated patient population includes those who have experienced prior postoperative wound complications, undergone reoperation due to fusion failure, or experienced prior radiation therapy [1]. A greater understanding of how overall health and comorbidities, extent of oncologic defects, prior radiation, bony quality, and functional status interact to affect patient selection criteria for spinoplastic surgery is vital [18,19,20].

## 2. Surgical Techniques, Indications, and Outcomes

### 2.1. Spinoplastic Techniques and Approaches

#### 2.1.1. Iliac Crest VBG

**Figure 1:**
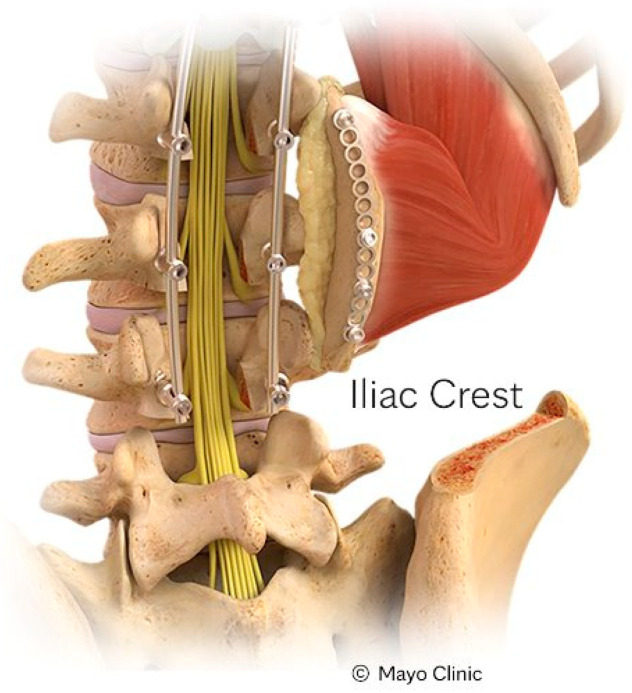
Illustation of the harvest and rotation of the IC-VBG pedicled on the quadratus lumborum muscle. The graft is tunneled under the multifidus and longissimus muscles and secured to the decorticated transverse processes of the lumbar spine. Reprinted with permission from Ref. [1]. 2024, used with permission of Mayo Foundation for Medical Education and Research, all rights reserved.

The iliac crest VBG (IC-VBG) to date has been the most commonly utilized VBG. Its bony segment is pedicled on the quadratus lumborum muscle, and it is typically indicated for posterolateral spinal reconstruction from T12 to S1 (Figure 1) (Table 1). However, a recent cadaveric feasibility study showed the potential of the IC-VBG to be utilized for anterior vertebral defects as well [21]. The surgical technique starts with a unilateral dissection over the posterior superior iliac spine [22]. An important structure to preserve during harvest is the cluneal nerve, containing branches which pass through the thoracolumbar fascia in the region of the iliac crest [23]. While the dorsal attachments of the iliac crest are dissected, the superior and deeper aspects of the quadratus muscle are preserved. Subsequently, blunt dissection of a tunnel under the multifidus and longissimus muscles is carried out from the transverse process of the lumbar spine laterally to the quadratus muscle. The bone graft is then released from the surrounding bone and rotated to the desired spinal level(s). The medullary surface of the newly created VBG is then placed in direct contact with the decorticated surface of the spine and is finally secured in place with titanium cables or miniplates and screws. It remains insufficiently studied how narrow the band of quadratus lumborum can be without compromising vascularity. In general, it is suggested to use at least a 2 cm attachment of quadratus muscle for each 5 cm length of bone [1,2,22].

#### 2.1.2. Scapular VBG

**Figure 2:**
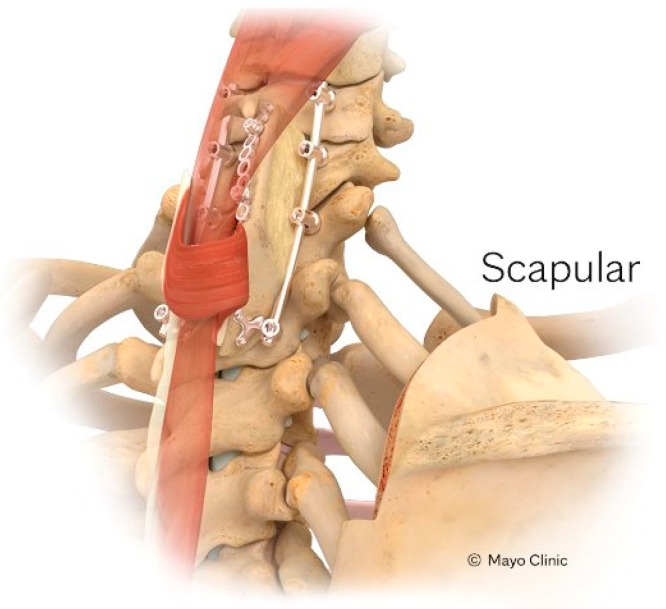
Depiction of the S-VBG pedicled on the rhomboid minor and trapezius muscles, harvested from the medial scapula. The graft is tunneled under the paraspinous muscles and secured to the posterior cervical or upper thoracic spine. Reprinted with permission from Ref. [1]. 2024, used with permission of Mayo Foundation for Medical Education and Research, all rights reserved.

The scapular VBG (S-VBG) is pedicled on the rhomboid minor and trapezius muscles and is used for posterior spinal reconstruction in its range of motion from the occiput to T8 (Figure 2) (Table 1). Care must be taken to preserve the descending branch of the transverse cervical artery during dissection [1]. The surgical technique starts with placement of the patient in the prone position and a midline incision as necessitated by the corresponding spinal surgery. Lateral dissection beyond the paraspinal muscles is carried out to access the medial border to the scapula. Once reached, the medial scapula is cut from either a superior or inferior angle while staying lateral to the descending branch of the transverse cervical artery. The subscapularis muscle is then divided from inferior to superior, halting before damaging the above-mentioned descending branch. At this point, the S-VBG is completely mobilized, remaining pedicled on the rhomboid and trapezius muscles. The graft is then tunneled under the paraspinous muscles and attached to the target bony lamina and spinous processes with miniplates and screws, and the newly fused construct is covered with bilateral flaps of paraspinous muscle [1,2,24].

#### 2.1.3. Rib VBG

**Figure 3:**
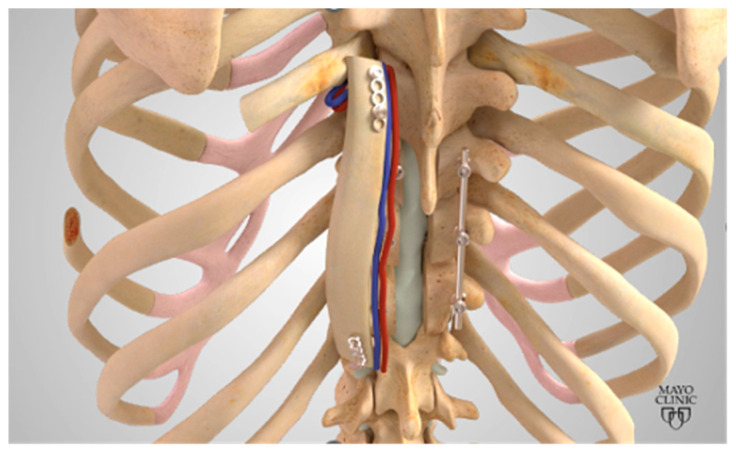
Visualization of the R-VBG pedicled on intercostal vessels. The rib is dissected free from intercostal muscle attachments and then tunneled and secured to the thoracic spine. Reprinted with permission from Ref. [1]. 2024, used with permission of Mayo Foundation for Medical Education and Research, all rights reserved.

The rib VBG (R-VBG) is pedicled on the intercostal muscles and is indicated for posterior spinal reconstruction in its range of motion from C6 to L5, depending on which rib is harvested (Figure 3) (Table 1). The 8th rib is typically targeted with vascular contribution stemming from perforators of the intercostal vessels. Important structures to preserve during harvest include the intercostal nerves and lung pleura. The surgical technique involves incision over the rib, with release of the intercostal muscle attachments. The rib and its vascular pedicle are then dissected from underlying pleura and inferior intercostal muscle attachments to protect subcostal vessels. Care must also be taken to separate the intercostal nerve from the vessels to prevent injury and subsequent neuropathy. The intercostal vessels are then clipped and incised, and the rib is cut proximally and distally, freeing the R-VBG. The rib is then tunneled through the paraspinous muscles, and its natural curvature is utilized to match the patient’s spinal curvature wherever it is placed. The rib is trephinated towards its final position facing the vertebral bone, and allograft is placed under it, creating a sandwich. The VBG sandwich is secured to the transverse processes of the vertebrae using 2 or 3 mm plating systems. Bubble testing can be utilized to rule out iatrogenic pneumothorax. Finally, rods are placed and aligned followed by closure with paraspinous muscle flaps [1,2,7,8,9,25].

#### 2.1.4. Occipital VBG

**Figure 4:**
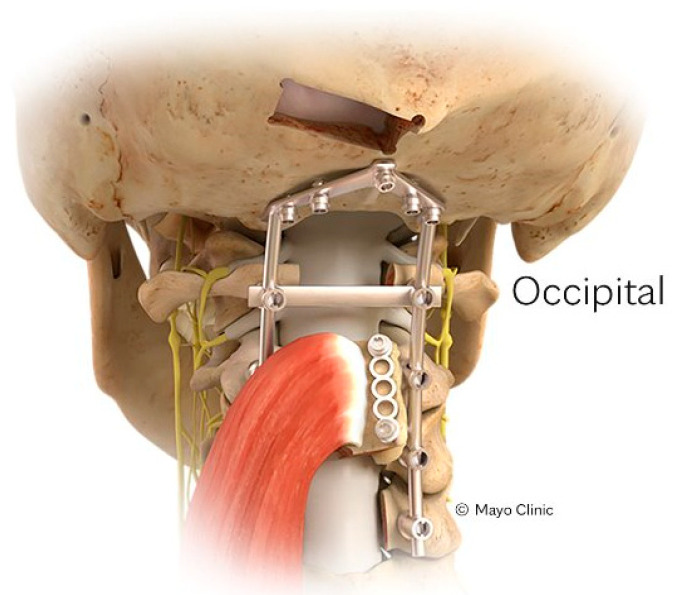
Demonstration of the O-VBG pedicled on the splenius capitis and semispinalis muscles. A segment of occipital bone is harvested subperiostally and secured to the cervical spine using fixation plates and screws. Reprinted with permission from Ref. [1]. 2024, used with permission of Mayo Foundation for Medical Education and Research, all rights reserved.

The occipital VBG (O-VBG) is pedicled on the splenius capitis and semispinalis capitis muscles and is indicated for posterior cervical spinal reconstruction in its range of motion from C1 to T1 (Figure 4). Typically, these muscles can only be sacrificed unilaterally to maintain stable rotation of the skull. The primary arterial contribution to Sharpey’s fibers is the deep cervical artery (Table 1). The surgical technique begins with the patient prone, and the neck neutrally positioned. A midline incision is made from the greater occipital protuberance to the target fixation point. First, the fascial plane between the trapezius and semispinalis capitis muscles is identified and dissected. A piece of the occipital bone is identified, bordered inferiorly by the foramen magnum and medially by the medial nuchal line. Dissection is then done subperiosteally around the occipital bone, with care not to disrupt the connections of the periosteum or semispinalis capitis muscle. The occipital bone is removed as one would perform a Chiari decompression. A portion of the occiput or the entirety of the bone may be removed while attached to a unilateral muscle depending on bone requirement. The VBG may even be taken vertically into the foramen magnum. The occipital bone is subsequently secured to the target site using fixation plates and screws [1,2,26].

#### 2.1.5. Spinous Process VBG

**Figure 5:**
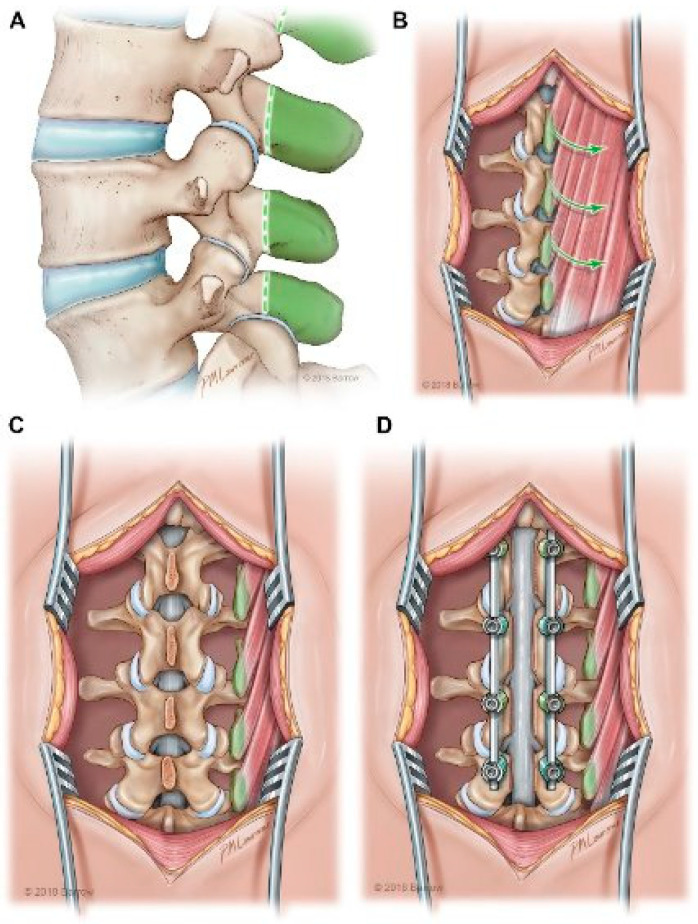
Illustration of SP-VBG harvest using adjacent spinous processes pedicled on intact paraspinal muscles. (**A**) Green shaded area depicts the bony part of the VBGs. (**B**) Illustration depicting the incision and exposure of the intact paraspinal muscle attachements. The arrows demonstrate the rotation of the VBGs once harvested. (**C**) Depiction of the spine once the VBGs have been mobilized. (**D**) Depiction of the spine once the SP-VBGs have been secured into place after decompression and hardware fixation. Reprinted with permission from Ref. [1]. 2024, used with permission of Mayo Foundation for Medical Education and Research, all rights reserved.

The spinous process VBG (SP-VBG) is indicated in the augmentation of arthrodesis in posterolateral fusion. It is pedicled on the paraspinal muscles and can cover deficits in its range of motion from the occiput to T8 by connecting the two adjacent spinous processes (Figure 5) (Table 1). Harvesting the graft begins with a midline incision over the area of interest and dissection of the superficial investing fascia of the paraspinal muscles. The facet joints and spinous processes of the vertebrae to be fused are exposed with care to leave the paraspinal muscles and periosteum intact for pedicle attachment. The contralateral superficial investing fascia is released and serves as the vascular pedicle for the SP-VBG. The spinous process graft can then be harvested without disrupting the muscular attachments. The soft tissue from the spinous process is removed, and both the graft and the transverse processes to be connected are decorticated to enhance bony fusion. Finally, the VBG is rotated and attached between the transverse processes in the PLF space. The graft is then stabilized with hardware and closed with the superficial investing fascia [26].

#### 2.1.6. Clavicular VBG

**Figure 6:**
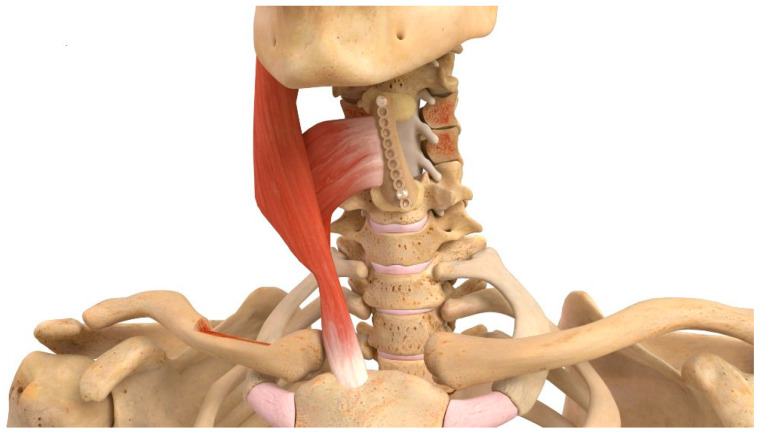
Diagram showing anterior cervicothoracic reconstrucion using a C-VBG pedicled on the sternocleidomastoid. The superior portion of the clavicle is mobilized and rotated to the cervical spine. Reprinted with permission from Ref. [1]. 2024, used with permission of Mayo Foundation for Medical Education and Research, all rights reserved.

The clavicular VBG (C-VBG) is pedicled on the sternocleidomastoid and supplied by the occipital and superior thyroid artery branches. It is indicated for anterior cervicothoracic spinal deficits in its range of motion from C2 to T2 (Figure 6) (Table 1). The surgical technique begins with exposure of the anterior cervical triangle via dissection of the platysma. The ventral aspects of C2-T2 are then dissected. The SCM is dissected to expose the sternal and clavicular heads. Subperiosteal dissection is then used to remove the ventral clavicle from the sternoclavicular joint while maintaining the inferior pectoralis and superior SCM attachments. The superior two-thirds of the clavicle are taken for the graft [27].

**Table 1:** Summary of VBG types used in spinoplastic surgery.

VBG Type	Blood Supply	Surgical Complexity	Complication Rate	Appropriate Clinical Scenario
IC-VBG	Quadratus lumborum	Moderate	Low	Posterolateral spinal reconstruction from T12-S1
S-VBG	Transverse cervical artery	Moderate-high	Low-moderate	Posterior cervical-thoracic reconstruction from the occiput to T8
R-VBG	Intercostal vessels	Moderate	Low-moderate	Posterior spinal defects with rib curvature alignment *
O-VBG	Deep cervical artery	High	Moderate	Posterior cervical reconstruction from C1 to T1
SP-VBG	Paraspinal muscles	Moderate	Low-moderate	Posterolateral fusion augmentation, can be utilized from the occiput to T8
C-VBG	Occipital & superior thyroid artery	Very high	High	Anterior cervicothoracic reconstruction from C2 to T2

* Arc of rotation to specific spinal levels depend on which rib has been selected for harvest.

### 2.2. Indications, Goals, and Identification of Suitable Candidates

Indications for the use of VBGs in spinal reconstruction primarily depend on a patient’s risk of developing pseudoarthrosis or other complications after spinal fusion surgery (Table 2). Selection of appropriate VBG(s) involves assessing the specific spinal levels targeted for fusion and identifying which segments might need additional structural support to ensure successful fusion and maintain spinal stability. Patients that have classically been identified as ideal candidates for a spinoplastic approach include those with previous fusion failures or existing pseudoarthrosis, those with congenital or severe spinal deformities, individuals undergoing extensive oncological resections that compromise spinal integrity, and patients who have elevated risks for infection or possess conditions that significantly impair bone healing [1,27,28].

Assessing risk factors for pseudoarthrosis is essential to optimize surgical outcomes after spinal fusion and identify patients that might benefit from a spinoplastic approach. A meta-analysis by Boonsirikamchai et al. showed that advanced age (>75) and smoking are significantly associated with increased risk of pseudoarthrosis after lumbar fusion [29]. Additionally, several comorbidities including vitamin D deficiency, diabetes, osteoporosis, and osteoarthritis have been identified to serve as positive predictors of symptomatic pseudoarthrosis after lumbar fusion. Studies also highlight that there is a direct correlation between symptomatic pseudoarthrosis and involvement of multiple spinal levels [30,31,32,33]. Specifically, Hoffler et al. found that cervical fusions involving 9 or more vertebrae, and thoracic or lumbar fusions involving four or more vertebrae significantly elevate the risk of pseudoarthrosis [30]. The study further confirmed that smoking and long-term steroid use universally increase the risk of pseudoarthrosis after fusion at all spinal levels [30]. Chronic opioid use, known for its negative impact on bone remodeling in vitro, has also been linked to development of pseudoarthrosis after fusion [32,34].

The Charlson Comorbidity Index (CCI) has shown promise in the identification of patients at risk for pseudoarthrosis for patients that underwent short-segment lumbar decompression and fusion, as higher index score correlated with higher rates of pseudoarthrosis [35]. Future research into the relationship between pseudoarthrosis rates after fusion and various comorbidity indices will be important in refining their value as a predictive tool in this space. Ultimately, awareness and careful consideration of these comorbidities and conditions during surgical planning are vital in accurately assessing a patient’s risk for pseudoarthrosis after spinal fusion surgery.

### 2.3. Contraindications, Risks, and Potiential Pitfalls

The literature on contraindications for the use of VBGs in spinal reconstruction remains limited. Active infection is widely recognized as an absolute contraindication to VBG use, as it poses increased risk for graft failure and systemic complications [36]. Osteoporosis and other bone disorders may serve as relative contraindications and should be carefully considered during patient selection [22]. Conversely, many factors that are traditionally viewed as relative contraindications, such as diabetes, radiation, prior surgery, etc. are in fact risk factors for pseudoarthrosis [37]. In these cases, the use of VBGs may be indicated rather than contraindicated, particularly in the setting of complex reconstruction need [37].

A lack of soft-tissue coverage may be considered a relative contraindication in select cases. However, this limitation is addressable though the utilization of local transposition muscle flaps which can provide robust soft tissue coverage [36,38]. Further research is warranted to better define absolute and relative contraindications for the use of a spinoplastic reconstruction approach.

Avaiable data on donor site morbidity for each VBG technique remains sparse. A review by Shin et al. found that donor site morbidity for R-VBGs is minimal, but includes chronic pain and functional limitation [39]. Although literature on pedicled IC-VBG donor site morbidity is limited as well, a review on free iliac crest bone flaps suggests that common donor site complications include change in sensation, chronic pain, and change in gait [40,41]. The change in sensation is thought to be secondary to injury to the lateral femoral cutaneous nerve during graft harvest [40].

It is important to note that technical pitfalls may occur during havest of VBGs, particulrarly if the vascular supply to the graft is disrupted during dissection. These risks highlight the importance of being thoroughly familiar with the anatomy and vascular pedicles unique to each graft type [1]. A lack of experience or unfamiliarity with these specialized techniques can increase the potential for intraoperative complications.

It is anticipated that the use of a VBG will increase operative time when compared with primary spinal reconstsruction. However, due to a lack of comparative studies, the extent of this increase, as well as the significance of other characteristics such as blood loss, complication rates, or recovery time are currently unclear. Notably, in cases where a free flap is already being considered, a pedicled VBG may reduce operative time and complexity [22]. While the adition of a VBG does introduce some degree of donor site morbidity, it appears that this morbidity is not significantly greater than that associated with free tissue transfer bone grafts [22,42].

### 2.4. Review of Published VBG Cases to Date

Several case series and reports have described patient characteristics and outcomes following spine surgery augmented with VBGs (Table 3). Reece et al. presented a series of 14 patients that underwent lumbar fusion surgery augmented by IC-VBGs. This cohort was identified as high risk for complications after spinal fusion due to comorbidities such as extensive osteomyelitis and discitis, and a history of multiple prior failed spinal fusions. All patients demonstrated successful graft fusion on imaging and had no procedure-related complications [22].

Additionally, Reece et al. reported using a S-VBG in a 56-year-old patient with a severe kyphotic cervical deformity, a 30-year smoking history, and previous neck radiation undergoing a C2-T4 fusion. This patient demonstrated significant improvement in his kyphosis at 3-month follow-up [24].

Another case by Reece et al. involved the use of an R-VBG in a 74-year-old paraplegic male patient undergoing fusion from T11 to the pelvis. He was considered high risk for pseudoarthrosis because of worsening pain and decline in sexual function following a T9-L2 fusion, and the development of a Charcot joint at the level of L2-L3. Postoperative imaging showed appropriate graft placement at the level of the Charcot joint, enhancing stability and promoting fusion at this segment that was at high risk for pseudoarthrosis [43].

Bohl et al. described two cases involving pedicled O-VBGs. The first, a 75-year-old female, experienced pseudoarthrosis and fractures following C2-to-pelvis fixation. She received an extension of fusion to the occiput augmented by O-VBG. Despite a postoperative surgical site infection requiring admission, imaging at 10 months confirmed successful fusion. The second patient, a 72-year-old female undergoing revision surgery for pseudoarthrosis at the C1-C2 level following a prior C1-C6 fusion, had risk factors including nicotine use, long segment fusion, and failed prior fusion. Thus, the use of an O-VBG to augment the procedure was indicated. Postoperative imaging showed successful graft fusion [44].

A case series published by Bohl et al. described a cohort of 4 patients that underwent spinal fusion augmented by SP-VBGs. The average age of the cohort was 66.5 years, and each patient had 2 or more risk factors for pseudoarthrosis. Although one patient had failure of fusion on imaging, all four patients had improvement in their functional status [26].

The use of a C-VBG was described in a case report that highlighted the technical challenges and complications with its use. A 69-year-old female with cervical myeloradiculopathy requiring multiple prior revisions underwent another revision with expansion of her corpectomy and augmentation with a C-VBG. She had multiple complications including a vocal cord palsy and shoulder pain secondary to dislodged hardware. She required another operation and symptoms ultimately improved with good evidence of graft fusion on 3 month postoperative imaging [27].

Another report by Reece et al. further illustrated the utility of VBGs in cases of extensive oncologic resections requiring postoperative radiation. In this report, a 17-year-old male underwent resection of a sacral myxopapillary ependymoma initially stabilized with titanium hardware. Due to the need for radiation therapy, another operation was planned to remove the titanium hardware and replace it with carbon fiber hardware and an IC-VBG for additional stability [45].

The versatility of pedicled VBGs was demonstrated by Shvedova et al. in a case report describing the use of bilateral simultaneous rib and iliac crest VBGs in a 68-year-old female patient with severe distal kyphosis. This patient had multiple prior operations to correct her kyphosis, which was severe enough to restrict oral intake and resulted in severe malnutrition. Despite these risk factors, the 3-month postoperative imaging showed appropriate placement and integration of all 4 VBGs [37].

Finally, Riasa et al. highlighted potential expanded indications of VBGs in resource-limited settings. The report described a 49-year-old male who underwent C3-C6 fusion augmented with an O-VBG, despite minimal risk for pseudoarthrosis. The VBG was utilized because this procedure was done at a center in Indonesia with limited access to alternatives such as bone allografts or free tissue transfers [46].

## 3. Discussion

Common themes emerging from the review of indications for a spinoplastic approach utilizing VBGs in spinal surgery emphasize the critical role of appropriate patient selection [9,22,24]. Across reviewed case reports, ideal candidates commonly presented with similar comorbidities and risk factors including prior fusion failures, significant spinal deformities, extensive oncological resections, high infection risk, advanced age, smoking, osteoporosis, and/or impaired healing due to systemic conditions or radiation. Additional factors influencing patient selection and risk stratification included extensive spinal involvement, chronic steroid use, and opioid use.

One particularly notable case report suggested expanding the indications for VBG utilization, especially in resource-limited settings lacking access to bone allografts or equipment for free tissue transfer [46]. As more data on the efficacy of VBGs accumulates, refining patient selection criteria will become increasingly essential. This may also enhance the predictive utility of established risk assessment tools, such as the CCI in spinoplastics, potentially improving preoperative decision making. Similarly, emerging technologies, including artificial intelligence driven risk stratification models, could further enhance patient selection and improve predictive accuracy regarding postoperative outcomes and complication risks, representing a promising future research direction.

From sacral reconstruction to stabilization of the cervical spine, the versatility and efficacy of VBGs across various complex clinical scenarios was demonstrated in this review. Among these, the IC-VBG remains the most frequently utilized VBG, supported by the most substantial body of evidence supporting its efficacy compared to other VBGs [22,28]. According to the literature, IC-VBGs consistently provided positive outcomes with minimal postoperative complications, even among high-risk patients suffering from osteomyelitis, discitis, and previous fusion failures [21,22,28,37]. Conversely, data on S-VBG use remains limited, although the initial outcome report appears promising [24]. Future investigation into the effectiveness and potential advantages of the S-VBG will clarify its place within spinal reconstruction.

O-VBGs showed excellent outcomes across 3 separate cases, demonstrating their utility even amid complications such as a postoperative infection [44,46]. R-VBGs showcased versatility when applied bilaterally and combined with IC-VBGs and demonstrated robust performance despite challenging anatomical positioning in a severely weakened spinal segment [37]. However, the C-VBG case report demonstrated significant procedural complexity and risk of complication, underscoring the importance of careful patient selection, preoperative planning, and ultimately refinement of surgical technique [27]. Lastly, the SP-VBG case series described good outcomes in all patients, despite graft integration failure in one [26].

Despite these promising results, spinoplastic surgery is still in its early stages, and significant challenges persist. The relative complexity of spinoplastic procedures requires specialized training and substantial institutional resources, potentially limiting the widespread adoption of these techniques. Spinoplastic surgery also requires buy in and multidisciplinary facilitation, which comes with its own logistical challenges. Furthermore, the current literature predominately comprises of case reports and small retrospective series, inherently limiting the generalizability and validity of the findings. Future prospective comparative studies will be critical in illuminating the scenarios which most warrant the consideration of spinoplastic surgical techniques employing VBGs, validating long term outcomes, and standardizing optimal patient selection criteria.

## 4. Conclusions

Spinoplastic surgery represents a new horizon in the field of spinal reconstruction, particularly beneficial for complex cases and patient populations that are susceptible to complications such as pseudoarthrosis and structural instability. Although data is limited, the consistent success and adaptability of VBG techniques to date underscores their crucial role in spinal care. Future research with a focus on comparative data will be necessary to refine patient selection criteria and foster broader application of this multidisciplinary approach to spine surgery.

## 5. Future Directions

Future research on spinoplastic surgery and the use of VBGs in complex spinal reconstruction should include controlled comparative studies alongside a control cohort of similarly complex spinal surgery patients to define clear indications and further validate the efficacy of VBG utilization. Additionally, there is a need to define standardized reporting protocols and outcome measures in these studies. Expanding research on some of the less frequently utilized VBGs will be important in clarifying their roles alone or in combination with other VBGs in spinal reconstruction. Treatment of posterior kyphosis deformity, primary VBG placement for scoliosis, as well as rib, functional muscle, and iliac crest free flaps are currently under investigation as potential reconstructive options. Further development and refinement of the utility of current comorbidity indices and artificial intelligence driven outcome prediction models will help guide patient selection and surgical planning. Lastly, exploring healthcare economics, access, and resource utilization, particularly in resource limited settings will be critical to the broader adoption and standardization of spinoplastic techniques at a global level.

## Figures and Tables

**Table 2:** Summary of indications for use of VBGs in spinoplastic surgery.

Indications
Previous fusion failures or pseudoarthrosis
Severe spinal deformities
Extensive oncological resections
Elevated infection risk
Impaired bone healing conditions (e.g., radiation therapy, diabetes, chronic steroid use, active smoking)
Extensive spinal involvement requiring augmented support

**Table 3:** Summary of evidence of spinoplastic techniques.

Authors (Year)	Study Type	Number of Patients	VBG(s) Utilized	Level of Evidence	Clinical Variables	Primary Clinical Outcomes
Reece et al. (2021) [22]	Case Series	14	IC-VBG	IV	IC-VBG in high-risk lumbar fusions	Successful graft fusion, no complications
Reece et al. (2021) [24]	Case Report	1	S-VBG	V	S-VBG for smoker with cervical kyphosis and prior radiation	Improved kyphosis, no graft complications
Reece et al. (2019) [43]	Case Report	1	R-VBG	V	R-VBG for paraplegic with prior fusion failure	Improved stability, no pseudoarthrosis
Bohl et al. (2021) [44]	Case Series	2	O-VBG	IV	O-VBG for failed prior cervical fusion	Successful fusion despite complications
Bohl et al. (2018) [26]	Case Series	4	SP-VBG	IV	SP-VBG for high risk lumbar fusion	Improved function; 1 fusion failure
Bohl et al. (2017) [27]	Case Report	1	C-VBG	V	C-VBG for cervical myeloradiculopathy	Fusion success and symptomatic improvement despite significant complications requiring reoperation.
Reece et al. (2024) [45]	Case Report	1	IC-VBG	V	IC-VBG for extensive oncolological resection	Imaging demonstrated succesful placement of VBG. Follow-up data limited.
Shvedova et al. (2024) [37]	Case Report	1	R-VBG, IC-VBG	V	Bilateral Rib & IC-VBG for severe kyphosis	Successful fusion, improvement of symptoms
Riasa et al. (2024) [46]	Case Report	1	O-VBG	V	O-VBG in resource-limited setting	Succesful fusion and imrpovement of symptoms despite limited resources

## Data Availability

No new data were created or analyzed in this study. Data sharing is not applicable to this article.
